# The efficacy of a range of hygiene measures for boot cleaning to protect natural vegetation from *Phytophthora cinnamomi*

**DOI:** 10.1038/s41598-023-32681-7

**Published:** 2023-04-10

**Authors:** Edward C. Y. Liew, Maureen Phelan, Keith L. McDougall

**Affiliations:** 1 Centre for Ecosystem Resilience, Australian Institute of Botanical Science, Royal Botanic Gardens and Domain Trust, Mrs Macquaries Rd, Sydney, NSW 2000 Australia; 2 Department of Planning, Industry and Environment, PO Box 733, Queanbeyan, NSW 2620 Australia; 3 Present Address: Department of Ecology, Environment and Evolution, School of Life Sciences, La Trobe University, Bundoora, VIC 3083 Australia

**Keywords:** Biological techniques, Ecology, Plant sciences, Ecology, Environmental sciences

## Abstract

*Phytophthora cinnamomi* is an oomycete found in the soil and capable of invading the roots of a wide range of host plants globally, potentially killing them and affecting the ecosystems they inhabit. This pathogen is often inadvertently dispersed in natural vegetation on the footwear of humans. A range of equipment is often provided or recommended to be carried for cleaning footwear in places where *P. cinnamomi* poses a threat to biodiversity. These are typically a brush for mechanically removing soil and/or a disinfectant for killing the pathogen. Despite their widespread use, to our knowledge, the majority of hygiene measures have not been experimentally tested for their efficacy. In the current study, we tested whether two types of brush and the two most widely used disinfectants (70% methylated spirits and benzalkonium chloride) were effective in removing the pathogen from boots. We tested the brushes and disinfectants in two soil types and two moisture levels. All hygiene measures were found to be better than doing nothing, although some were only effective with sandy or dry soils. Benzalkonium chloride was largely ineffective as a spray but highly effective when used in a footbath. Brushing did not improve cleaning when used with 70% methylated spirits. None of the hygiene measures was completely effective for cleaning boots that had been in wet loamy soil. Our findings have important implications for management of this threat because some recommended hygiene practices are not doing what they claim.

## Introduction

*Phytophthora cinnamomi* Rands is one of only three plant pathogens listed as the 100 world’s worst invasive alien species in the Global Invasive Species Database^1^. While evidence suggests it originated in South East Asia and parts of east Asia^2,3^, this oomycete has a global distribution today where it not only causes economic losses in a wide range of agricultural and horticultural productions^4–6^, but has devastating impacts on native ecosystems^5,7–10^. As a plant pathogen, *P. cinnamomi* has a remarkably broad host range^11^, estimated to cause diseases in over 5000 plant species^4,6^.

In Australia, dieback or root rot caused by *P. cinnamomi* is listed federally as a ‘Key Threatening Process’ under the *Environmental Protection and Biodiversity Conservation Act* 1999 and in various State legislatures, highlighting its enormity as a threat and the urgency for managing its effects. Over 2000 native species have been estimated to be susceptible to the pathogen in southern Western Australia alone, some of which typically form the dominant structural component of natural ecosystems^12^. The pathogen may also indirectly affect faunal communities by changing vegetation structure or removing food plants^13^. At least 135 species at risk of extinction in Australia may be directly or indirectly affected by *P. cinnamomi*^14^. Impacts on native plants and vegetation have similarly been noted in the UK, Europe, South Africa and North America^5^. *Phytophthora cinnamomi* is yet to fully occupy its potential niche both globally^15^ and at a local scale, with indications of its continual spread, the slow but emerging knowledge of species susceptibility, the challenges in effective management and the exacerbation of the impact of this disease by other ecological threats such as drought, fires, pests and other diseases^16^.

*Phytophthora cinnamomi* infects and colonises the roots of susceptible plants, disrupting their fundamental capacity of water and nutrient uptake. It spreads within plant tissue in its vegetative form, the mycelium, which also enables it to progress from host to host through root contact. Under moist conditions, the mycelium produces sporangia which release zoospores into the soil environment^4^. These short-lived zoospores are motile and have the ability to detect and locate actively growing roots of nearby hosts via chemotactic response to root exudates^6^. These active forms of dispersal and growth facilitate short distance disease spread. *P. cinnamomi* produces chlamydospores, asexual survival or resistant propagules, often in abundance in association with infected host tissue^17^. These chlamydospores are believed to allow the pathogen to persist in the soil and infected tissue under harsh conditions (e.g. dry and hot periods), but will readily germinate once environmental conditions become conducive for vegetative growth and production of the motile infective propagules^18^, thus facilitating the perpetuation of an asexual disease cycle.

In addition to active dispersal over short distances in soil and water, *P. cinnamomi* may be spread over longer distances through the passive movement of contaminated soil by humans and other animals, as well as run-off after storm events^5,16^. While non-human animals spread contaminated soil within home ranges on feet and even faeces^19^, humans have the capacity to spread the pathogen over much greater distances through contaminated footwear, tools, equipment, vehicles (both occupational and recreational) and saddle horses. Furthermore, anthropogenic spread is not necessarily confined to one site or geographical region and there is good evidence to suggest *P. cinnamomi* has been spread to even remote areas through human mediated dispersal, e.g. to the Greater Blue Mountains World Heritage Area in NSW, Australia^20^. Once the pathogen is introduced into an area of natural ecosystem, it is practically impossible to eradicate^21^. Hence, regardless of how infrequent or small the amount the movement of contaminated soil may be, the risk of disease spread is arguably significant over time.

Managing *Phytophthora* diseases in natural ecosystems presents a particularly challenging undertaking due to the highly limited management tools available, unlike in production systems where the introduction of horticultural practices, genetic resistance and agrochemicals form a routine suite of tools in the Integrated Pest Management (IPM) armoury. While eradication is seldom feasible, the guiding principle in managing *P. cinnamomi* has largely been based on the containment of the pathogen where it is present, preventing its dispersal and introduction to new locations, thus reducing its potential impact. Hygiene practices are the most practical on-ground disease management tools available for spread mitigation where human access cannot be restricted or completely prevented. These measures commonly include cleaning and disinfecting anything that comes into contact with soil. While the dispersal of pathogen propagules on vehicles is generally confined to well-defined roads and of limited bounds, hikers can reach remote areas and may spread the pathogen into previously pristine environments. Hygiene options for hikers are varied but typically involve removing soil, mud and organic particles from boots and disinfecting with one of a range of disinfectants. Measures including scrubbing boots with a manual brush, scrubbing on a fixed boot scrub, spraying or soaking boots in disinfectant tubs, or stepping onto a disinfectant footbath or mat, have been recommended as part of best practice management guidelines in various jurisdictions globally^22–29^ where the threat of soil borne pathogens, including *Phytophthora*, exists. The efficacy of these hygiene measures and their effectiveness in managing disease spread, however, is not well studied. While there is a plethora of studies on the utility and efficacy of footwear hygiene practices in veterinary and animal husbandry settings^30–35^ as well as several studies in hospital^36^, nursery^37^ and passenger transport^38,39^ scenarios, there are hardly any data on the efficacy of footwear hygiene in natural vegetation areas. Notably one study in New Zealand^40^ demonstrated that the soil borne Kauri pine pathogen, *Phytophthora agathidicida*, was effectively eliminated from boots contaminated with soil containing the pathogen by spraying either of two commercial disinfectants, Phytoclean™ (a.i. benzalkonium chloride, quaternary ammonium compound, BAC) or TriGene™ (II) Advance (a.i. halogenated tertiary amines), without the prior removal of soil particles from the boots. Elsewhere, we expect, recommended hygiene measures for hikers are assumed to be effective because they have been used in other settings. But are those assumptions reasonable?

The success of a hygiene program in preventing the spread of pathogens by humans will depend on three variables: the likelihood of pathogen spread, the effectiveness of hygiene measures, and compliance. These variables are interconnected and so for instance, a low likelihood of spread will mean that hygiene measures and compliance are less critical to success; highly effective hygiene measures will be worthless if there is low compliance and conversely, high compliance but ineffective hygiene measures will not prevent spread. In the case of the pathogen *Phytophthora cinnamomi*, the implications of a failed hygiene program where the risk to natural vegetation is high, are immense.

While hikers are regarded as being important vectors of pathogens in natural areas (e.g. *Phytophthora ramorum* in the US^41^, *P. cinnamomi* in Australia^42^, *P. agathidicida* in NZ^43^) but the magnitude of the threat of pathogen spread by hikers has rarely been quantified. Tjosvold et al*.*^44^ found that detections of *P. ramorum* from hiker boots in field conditions in California ranged from 0 to 95% depending on rainfall. Pau’Uvale et al*.*^43^ recovered five *Phytophthora* species from an undefined number of boots in bushland area near Auckland, New Zealand. If these pathogens are a threat to environmental assets in an area frequented by hikers, a high likelihood of spread on boots should be assumed.

In the current study, we report on experiments testing the efficacy of hiker hygiene equipment typically used in the field: (1) hygiene kit comprising a handheld brush and a disinfectant spray bottle; (2) fixed boot brush or disinfectant foot bath; and (3) commercial hygiene station with fixed brushes and disinfectant spray.

## Methods

### Experimental materials

#### Boots

**Figure 1:**
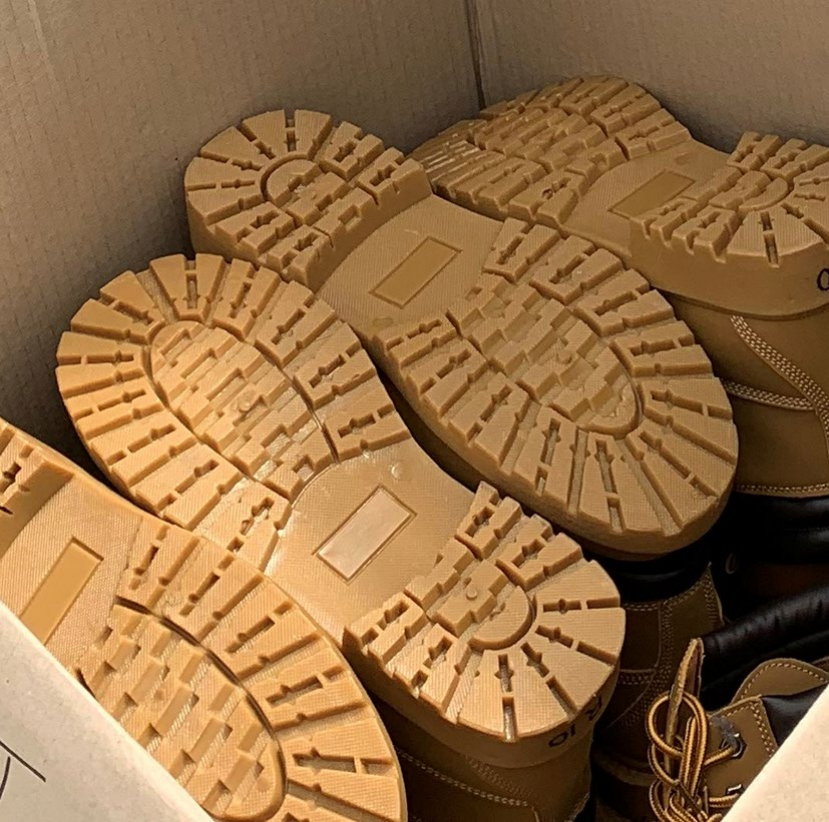
Boots used in the study showing the deep and complex tread pattern of the sole.

Hiking boots were purchased from Big W (Woolworths Group, Australia). For the total number of boots required, it was not possible to obtain all boots of the same size, hence men’s size 9 (European size 43) and size 10 (European size 44) were purchased. The soles were made of a blend of rubber and foam (Vibram^®^) with a sole thickness of 35 mm/15 mm (heel/ball) and a complex tread 3 mm deep (Fig. 1).

#### Handheld brushes

**Figure 2:**
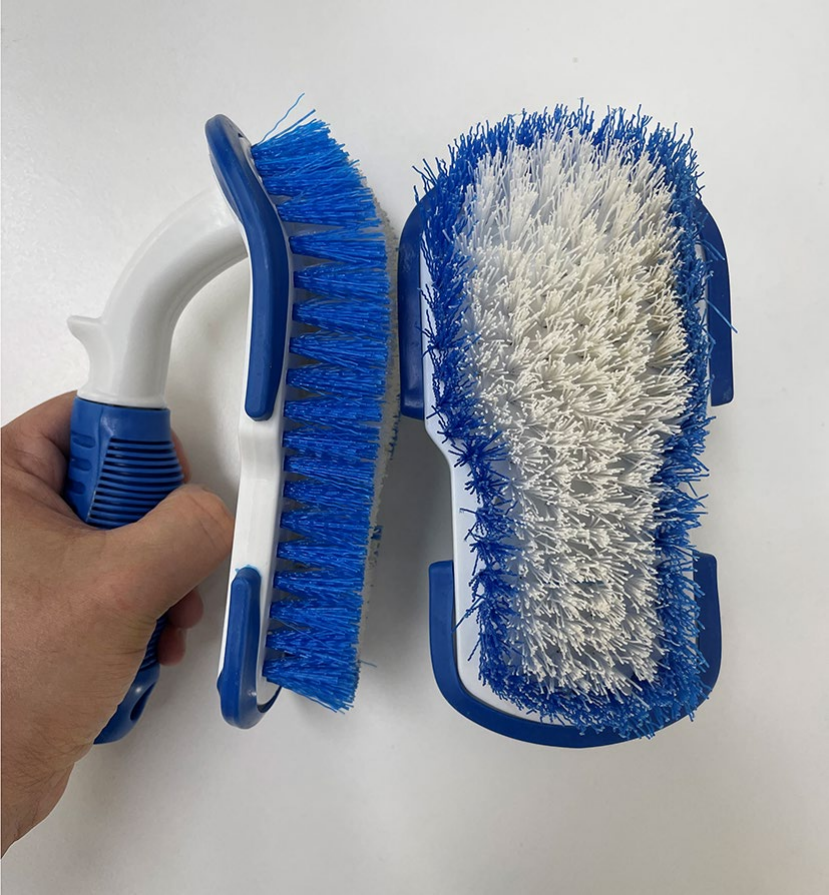
Durable handheld brushes selected for use in the study.

Handheld durable brushes made of PET plastic with firm bristles were selected based on their durability, as indicated by any visible signs of defects after more than 8 cycles of bleaching and autoclaving. The bristle length was 25 mm, with an approximate brushing area of 110 cm^2^ and a density of approximately 35 bristles cm^−1^ (Fig. 2).

#### Fixed boot brush

**Figure 3:**
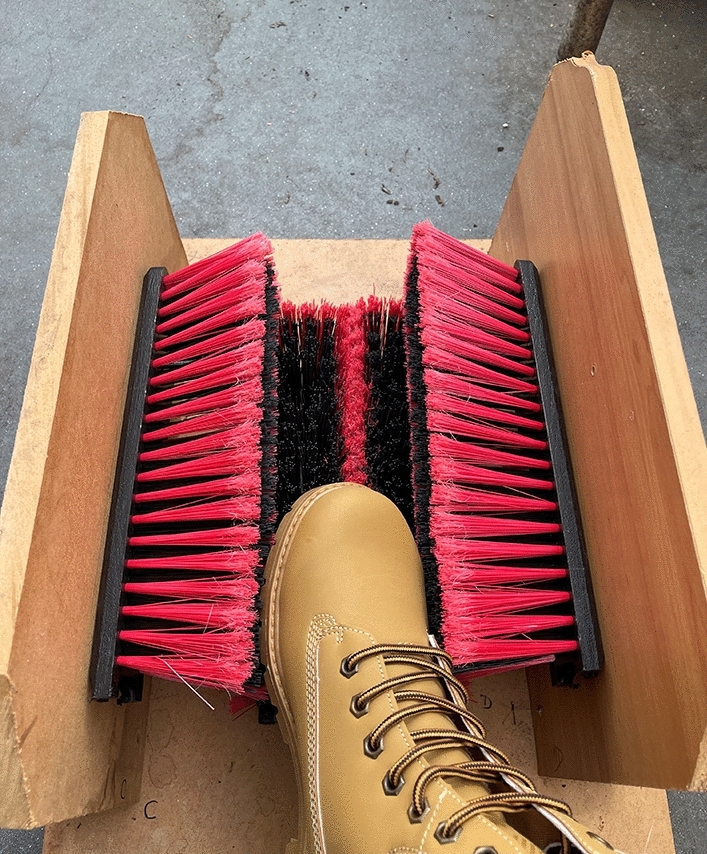
Fixed boot brush device, constructed from broom heads bolted onto timber sheets, used in the study.

The fixed brush was constructed from two broom heads. The broom heads were cut in two and fixed to timber sheets so that each boot would fit between the brushes with some resistance, facilitating brushing of the sides as well as the sole. The brush bristles were made of firm PET plastic measuring 80 mm in length. Each rectangular brushing surface (half broom head) was approximately 270 cm^−2^ (30 cm × 9 cm) with two types of bristles: thinner fibre around a 1 cm margin of the brushing surface (45 bristles cm^−1^) and thicker fibre in the centre (18 bristles cm^−1^) (Fig. 3). A bespoke fixed foot brush was used rather than a commercial brand to facilitate dismantling, cleaning and disinfecting after each test.

#### Soils

Two commercial soils were obtained, referred to as loam and sand. The loam was purchased from Grange Growing Solutions (NSW, Australia) and amended to a final composition of composted grade pine bark:coir:propagation sand:perlite (11:2:2:5; pH 5.3–6.5; EC < 2.2). The sand was unamended course grade river sand from Bastion (Australia; pH 6.0–6.5). All soils were spread thinly on plastic tubs to about 3 cm thick and sun dried for 4–8 h with regular turning. Four soil/moisture types were included in the study: Dry Loam, Dry Sand, Wet Loam and Wet Sand.

Percentage moisture content for each soil type was measured by obtaining a sample of the experimental soil, slow oven drying at 80–90 °C, with initial weight measured and then daily thereafter until weight stabilised (c. 8 days). This was replicated 3 times for each soil type and the mean recorded (Dry Loam 0.07%; Dry sand 0.05%; Wet Loam 0.31%; Wet Sand 0.12%).

#### Inoculum

Cultures of *Phytophthora cinnamomi* (accession RBG W1324) were revitalised and cultured from long term storage under sterile deionised water. Once sufficiently grown, they were passed through lupin seedling baits (*Lupinus angustifolius*) in a baiting cylinder containing sterilised soil and the culture (as 1 cm^2^ agar plugs). Infected lupin stem and root material were aseptically cut into 5 mm sections and plated onto V8 agar (20% clarified Campbell’s V8 juice, pH 6.5–7.5 adjusted with calcium carbonate). The cultures were incubated in the dark at 24 °C for 4 days prior to subculturing onto more plates. The subcultures were incubated for 2 weeks and used to seed inoculum mix which consisted of commercially available millet, wheat bran, and river sand (1:1:1 by volume) in autoclavable polycarbonate jars (Nalgene, 500 mL with 120 mm diameter). The jars were filled up to a third full then shaken and rolled for consistency. The mix was then moistened with c. 15 mL of deionised water prior to autoclaving twice at 121 °C. Six 1 cm^2^ plugs of culture were placed into each jar followed by incubation in the dark for three weeks. The jars were subsequently examined aseptically for mycelial growth throughout the millet-bran-sand mix before being pooled and mixed in a surface sterilised tub prior to infesting soil for the experiments.

#### Buckets

Labelled plastic buckets of 9.3 L capacity were used to contain and transfer individual boots after each experimental treatment.

#### Spray bottles

Plastic spray bottles of 500 mL capacity were used for the disinfectant spray treatments. The nozzles were adjusted to allow for a medium fine conical spray of approximately 1.3 mL of liquid per full spray.

#### Disinfectant footbath

**Figure 4:**
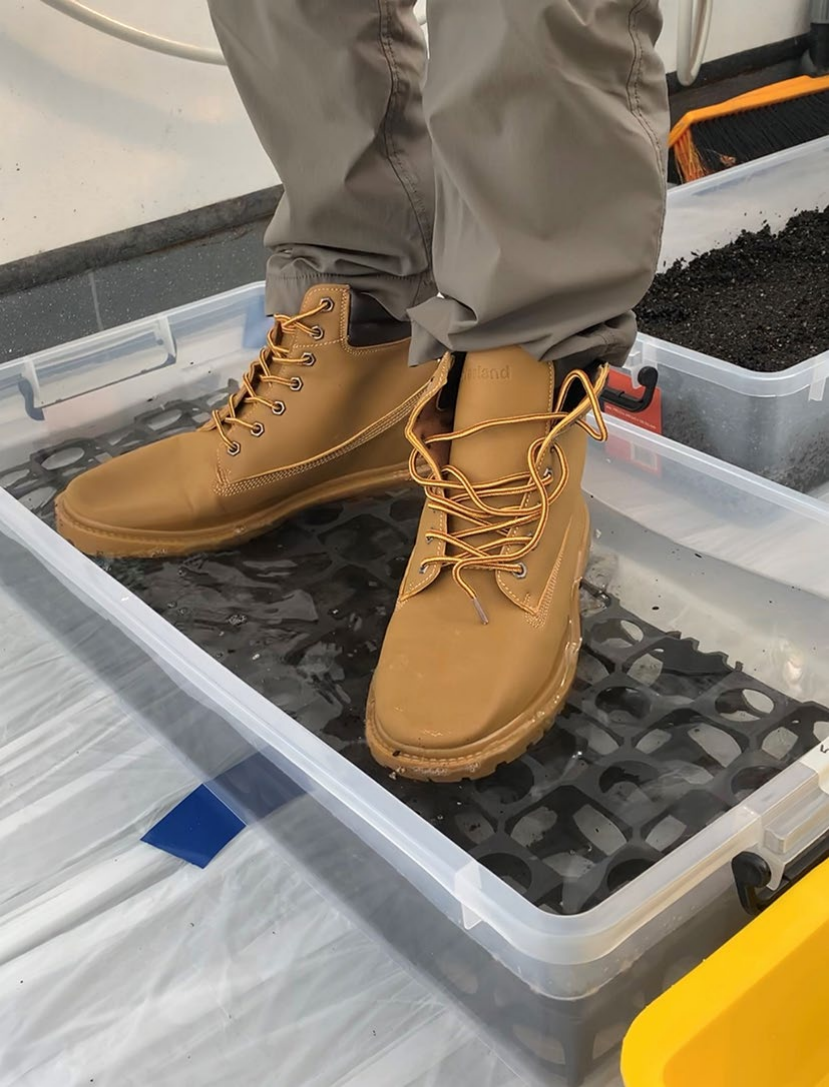
A volunteer standing in a plastic disinfectant footbath lined with drainage cells.

Shallow plastic tubs of 38 L capacity (wdh: 740 cm × 35.5 cm × 14.5 cm) were used as disinfectant footbaths filled with 14 L of disinfectant, lined with plastic nursery drainage cells (5 cm thick) to stand on while allowing the soles to be fully in contact with the solution (Fig. 4).

#### Disinfectants

Disinfectants used in this study were methylated spirits (100% denatured, Diggers) diluted with deionised water to 70% as spray solution, and Phytoclean™ (100 g L^−1^ BAC) diluted with deionised water to various concentrations according to the label and manufacturer’s recommendations: 2% solution in manual spray bottles, 10% solution in footbaths and 10% solution as boot spray in the hygiene station.

#### Plastic tubs

**Figure 5:**
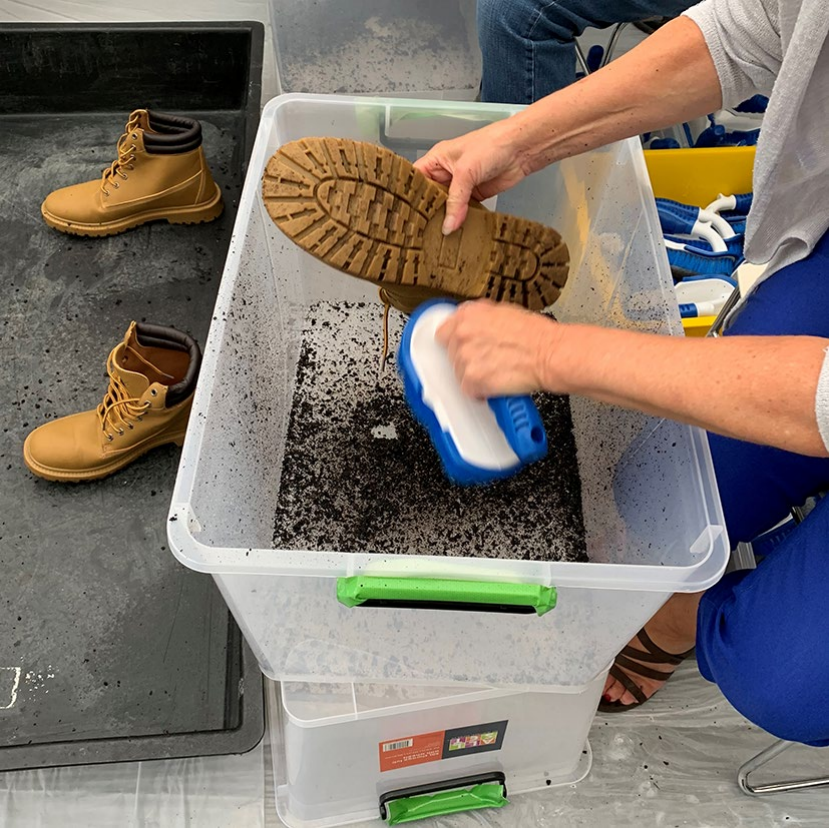
A volunteer cleaning a hiking boot with a handheld brush. Soil was dislodged from the boot and collected in a clean plastic tub and the cleaned boot was then placed in a bucket for subsequent pathogen processing.

A range of plastic tubs were used to contain soil dislodged during cleaning using handheld brushes (Fig. 5).

#### Hygiene station

**Figure 6:**
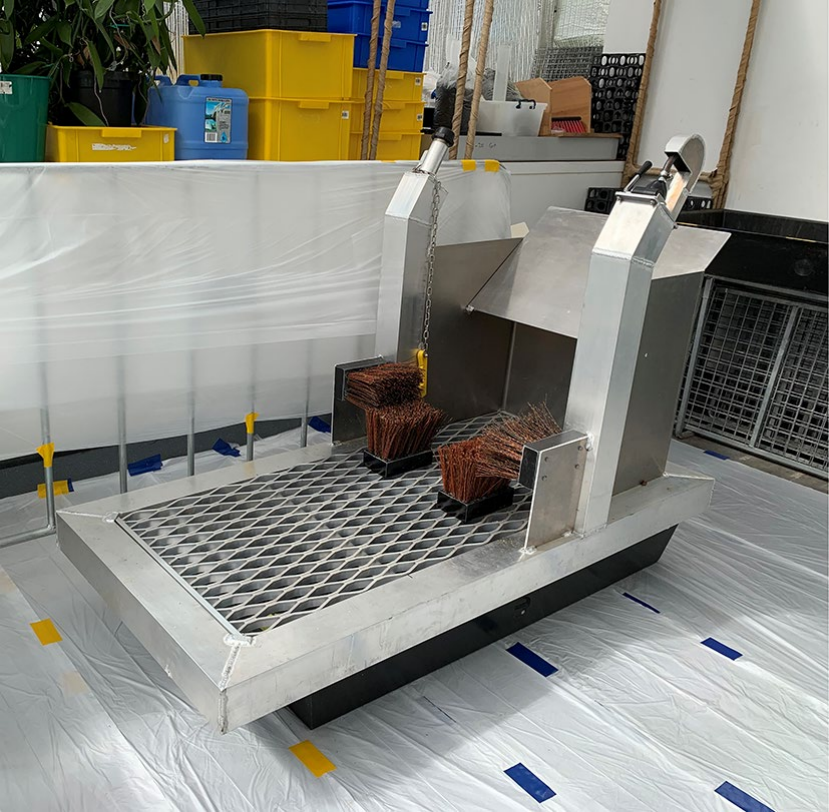
Hygiene station used in the study. The whole machine was placed on top of a large sturdy tub to catch soil particles through the grate. Note the fixed boot brushes, and the disinfectant spray lever on the right.

A commercial hygiene station was used for the study. Here we describe only the components of the station rather than naming the brand as all available stations of this nature use similar methods for cleaning. The station consisted of a stage with open metal grating on which a set of four detachable brushes were affixed, positioned for optimal brushing of the bottom and sides of each boot, and handles on either side of the station to aid user balance. The brush bristles were made of thick firm PET plastic measuring 125 mm in length. The brushing surface of each of the four rectangular brushes was approximately 170 cm^−2^ (17 cm × 10 cm) with 8–10 bristles cm^−1^. After completing the brushing action, users rested each boot on a footrest in between the brushes and applied disinfectant sprays to the sole using a hydraulic hand lever (Fig. 6).

#### Volunteers

Volunteers for the series of experiments were recruited from the Royal Botanic Gardens and Domain Trust (the Trust) volunteer program by advertising in their webpage, requesting assistance with minimal description of the tasks. These volunteers all underwent an induction and introduction session, and formally registered as compliant with the Trust Volunteer Policy. A total of 34 male and female volunteers were recruited, ranging in age from 20 to 70 s. Very few performed many replicates and most only a few. To enable their inclusion as a variable in the analysis, volunteers were classified as either male or female and either over or under 60 years of age. The experiments were conducted in accordance to the Guidelines and Policy of the Trust. In addition to approval from the Trust to conduct the experiments, the explicit consent from each volunteer was obtained.

### General experimental procedures

All experiments were conducted in a 5 m × 3.6 m greenhouse lined with double layers of plastic drop sheets prior to each set of experiments, after which they were sprayed with 70% ethanol and disposed of before replacing with fresh sheets.

All boots, brushes, buckets and tubs were washed and disinfected prior to each experiment. The boots were rinsed in tap water, steam pasteurised and air dried. The brushes were rinsed with tap water with the aid of disposable skewers to dislodge soil particles, then soaked in 1% hypochlorite solution (household bleach) for 20–30 min and rinsed. The same disinfecting procedures were carried out for the detachable brushes (from the hygiene station and fixed boot brush). The buckets and tubs were all manually washed with tap water, surface sterilised with 70% Ethanol and air dried. To ensure that no pathogen contamination was present at the commencement of each experiment, 20 boots and 20 brushes were arbitrarily chosen and tested for the presence of *P. cinnamomi* prior to any experimental treatments.

Infested soil was prepared by placing 13 L of dried soil and 500 mL of inoculum mix (prepared as described above) in a large plastic tub and mixing it thoroughly with hand trowels prior to the experiments. For wet soil treatments, 3 L of deionised water was added to each soil-inoculum mix and homogenised. Soils containing the inoculum were stirred frequently during the experiments to prevent compaction.

**Figure 7:**
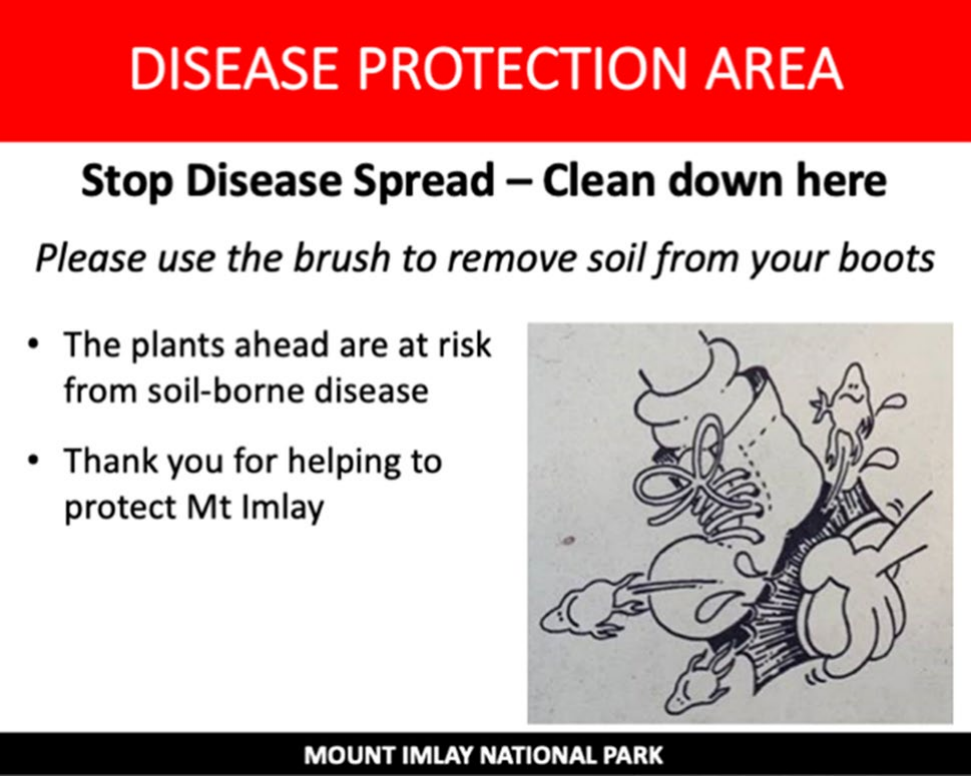
Hygiene sign from a track in Mount Imlay National Park, NSW, shown to volunteers prior to boot brushing in the current study.

**Figure 8:**
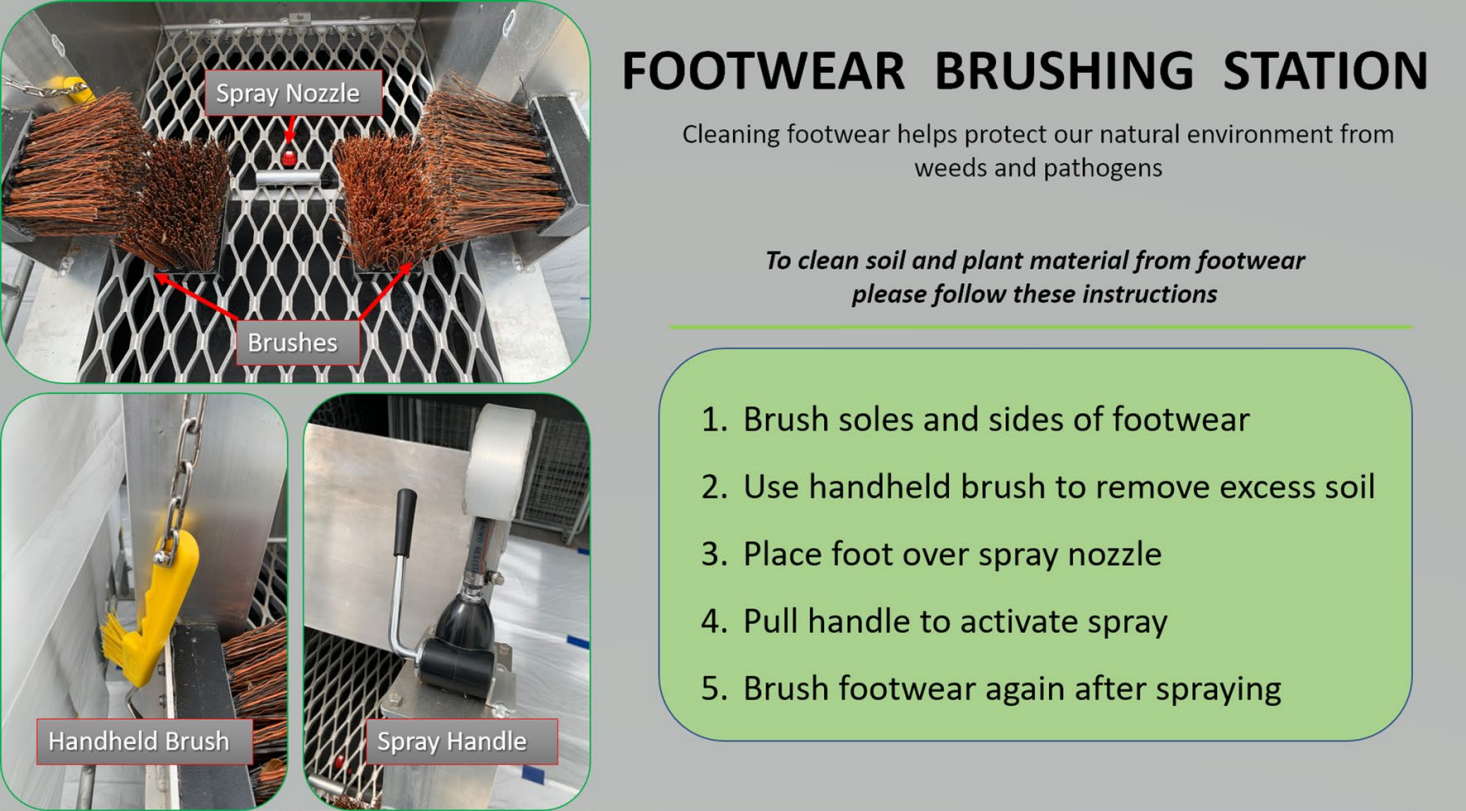
Hygiene station instructions shown to volunteers during the study modified from signage in a NSW national park with a similar station.

At the start of each experiment, the volunteers were given information about the pathogen, its impact and mode of dispersal, and shown a hygiene sign from a local national park (Fig. 7). They were then shown the equipment provided and given general information about the experimental procedure: i.e. (a) the purpose of hygiene was to clean the boots; (b) the brushes were designed to remove soil; (c) the disinfectant was used to kill the pathogen rather than to remove soil and so complete coverage of the sole was important. For the experiments involving a commercial hygiene station (referred to as Equipment 3 below), they were also shown information about how to use the equipment (Fig. 8 as used in field situations). However, for all experiments they were not instructed how long to brush or how many squirts of spray to apply.

### Experimental designs

A series of experiments were designed and conducted based on hygiene tools and devices, either fixed or portable, generally available in field scenarios (including depots and greenhouses). These were Equipment 1: hygiene kit comprising a handheld brush and a disinfectant spray bottle (1a: disinfectant = methylated spirits; 1b: disinfectant BAC); Equipment 2: fixed boot brush or disinfectant foot bath; and Equipment 3: commercial fixed hygiene station with fixed brushes and disinfectant spray.

#### Efficacy of Equipment 1 (field hygiene kit)

These experiments were designed to simulate a field situation where a hiker has a portable hygiene kit containing a brush and disinfectant spray bottle, and instructed to clean their boots before proceeding past a certain point. To expose the boots to the pathogen, a volunteer put on a pair of boots and stamped in contaminated soil for 30 s. Each boot was then removed for one of six experimental treatments: (i) no hygiene measure (control); (ii) brushing using a handheld brush; (iii) spraying the sole with 70% methylated spirits; (iv) spraying the sole with 2% BAC; (v) brushing followed by spraying with 70% methylated spirits; and (vi) brushing followed by spraying with 2% BAC. Twenty replications were performed for each treatment (i.e. 20 boots per treatment). The time spent brushing the boot and the number of disinfectant squirts applied by the volunteer were recorded. After the hygiene treatment, each boot was placed in a pre-labelled bucket for subsequent testing for *P. cinnamomi* (as described below). The experiments for the four soil/moisture types were performed 1 week apart, reflecting the limited capacity of the laboratory to process samples.

For equipment 1 we initially instructed volunteers to brush their boots while they were being worn. We observed inconsistencies between volunteers with respect to their flexibility and hence brushing method, coverage and rigour. The results of this were inadvertently informative for hiker management in general and are reported below under “Hygiene metrics and volunteer observations”.

#### Efficacy of Equipment 2 (fixed boot brush and disinfectant footbath)

These experiments were designed to simulate a hygiene device comprising a fixed boot brush or a footbath or both. To expose the boots to the pathogen, a volunteer put on a pair of boots and stamped in contaminated soil for 30 s. The volunteer then proceeded directly to one of four treatments: (i) no hygiene measure (control); (ii) a fixed boot brush positioned in a trough to catch soil dislodged from boots; (iii) a footbath of 10% BAC; and (iv) the fixed boot brush followed by the footbath containing 10% BAC. Volunteers spent 30 s in the footbath. The number of passes through the fixed brush by the volunteer was recorded for each replicate rather than the time spent brushing because volunteers would often pause during brushing making timing difficult. After the hygiene treatment, the boots were removed and each boot placed in a pre-labelled bucket for subsequent testing for *P. cinnamomi* (as described below). The footbath was replaced between treatments with a new tub containing fresh BAC solution. Twenty replications were performed in pairs for each experimental treatment (i.e. 10 pairs or 20 boots per treatment). The order of boots was recorded because the foot brush and footbath were clean for the first pair of replicates but became progressively dirtier as the experiment proceeded; it was thought that the likelihood of cleaning or disinfecting boots might decline during the experiment. The experiments for the four soil/moisture types were performed one week apart.

#### Efficacy of Equipment 3 (commercial hygiene station with fixed brushes and disinfectant spray)

These experiments tested a commercially available station where hikers brush their boots on fixed brushes and then spray the soles with 10% BAC, applied with a hydraulic pump. To expose the boots to the pathogen, a volunteer put on a pair of boots and stamped in contaminated soil for 30 s before directly proceeding to one of two treatments: (i) no hygiene measure (control); and (ii) the commercial hygiene station, where each volunteer stepped onto the station and brushed their boots one at a time. After brushing, the volunteer placed the boots one at a time on the footrest with the sole raised to face a sprayer. The number of sprays applied using a manual pump lever and the % sole coverage of BAC were estimated for each boot (replicate). Immediately after stepping off the station, the boots were taken off and each boot placed in a pre-labelled bucket for subsequent testing for *P. cinnamomi* (as described below). Twenty replications were performed for each experimental treatment. The experiments for dry and wet soils were performed one week apart.

### Additional experiments

#### BAC concentration

Two BAC concentrations (5% and 10%) were tested using a footbath and handheld spray bottles in contaminated wet loam to assess whether the concentrations recommended on the label for different application techniques gave different outcomes. Twenty boots were stamped in contaminated wet organic soil for 30 s and then given the following treatments: (i) no hygiene measure (control); (ii) standing in a footbath for 30 s in 10% BAC; (iii) standing in a footbath for 30 s in 5% BAC; (iv) spraying boot soles with seven squirts for 100% coverage with 10% BAC; and (v) spraying boot soles with seven squirts for 100% coverage with 5% BAC. The average number of squirts of disinfectant applied by volunteers was seven.

#### Field trial

**Figure 9:**
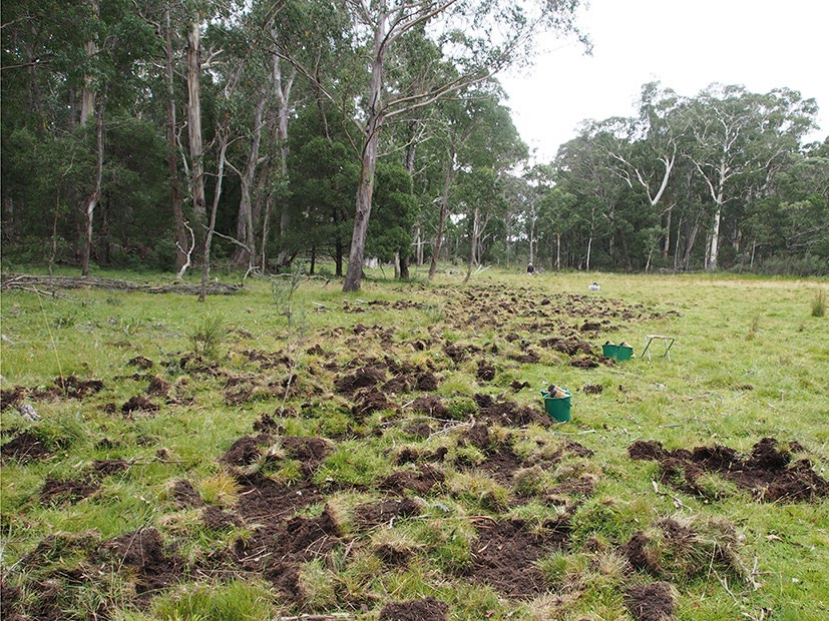
Nunnock Swamp Nature Reserve, NSW, where soil samples were obtained for *Phytophthora* testing from holes dug by pigs. The exposed clumps of soil allowed easy collection of replicated samples on boots using a protocol similar to the one used in the greenhouse experiments.

A field trial was set up in an area known to contain *P. cinnamomi* to assess (1) how easily the pathogen may be spread under field conditions and (2) whether the amount of inoculum used in the experiments was broadly representative of field conditions. Nunnock Swamp Nature Reserve is in south-eastern New South Wales at an elevation of about 1000 m. It comprises tall open forest surrounding a large swamp of shrubs and sedges. The soils there are highly organic and slightly peaty, similar in texture to the loam soil used in the experiments described above. *P. cinnamomi* has been detected in several places at the swamp margin. At one such place, soil samples (approx. 250 g) were collected in 20 locations where pigs had exposed the soil in search of plant tubers (Fig. 9) and sealed in labelled plastic bags. At each of these locations, a volunteer put on a pair of the same boots used in the experiments described above and stomped in the soil for 30 s before placing each boot in a bucket. The soil at the time of collection was damp and adhered easily to the boots. Soil from 40 boot samples was brushed and washed into ziplock bags as described above for the experiments and, with the 20 soil samples taken at the pig digging locations, taken for testing in the laboratory as outlined below.

### Testing for *Phytophthora cinnamomi*

For all the experiments, each boot after every treatment, including positive and negative controls, was placed in a sterile bucket for processing to determine the presence of *P. cinnamomi.* A sample for testing was obtained by meticulously removing remaining soil on each boot with the aid of a disposable toothbrush, a disposable skewer (where necessary) and a squirt bottle of deionised water, and collecting the soil–water sample in a pre-labelled ziplock bag. Each bag was then transferred to the diagnostic laboratory where routine soil testing for *Phytophthora* was conducted.

Approximately 50 mL of sterile loam (for induction of sporangium production and zoospore release) was added to each sample bag, propped up in a plumbing pipe and flooded with deionised water. Positive (addition of agar plugs of *P. cinnamomi* culture) *and* negative controls were included for each batch of testing. Pre-germinated blue lupin seed, *Lupinus angustifolius* (a garden variety purchased from Rockfield Pty Ltd, Tasmania, Australia), were suspended on the soil–water slurry and incubated for 7 days at ambient temperature, after which the germinated lupin seedlings were harvested. [The use of blue lupin seedlings in the experiments is a routine protocol in the Royal Botanic Gardens Sydney and in compliance with the scientific research guidelines of the Trust.] Total eDNA was extracted from the distal 10 mm of the harvested lupin radicles using the FastDNA Kit (Q-biogene Inc., Irvine, California, USA) according to the manufacturer’s instructions. The presence of *P. cinnamomi* DNA detection was based on specific PCR where the ITS and Ypt1 were amplified using primer sets and PCR conditions described in^45^ and^46^, respectively. Presence of the specific amplicon, as observed via electrophoresis and staining (SYBR Safe, Invitrogen, Thermo Fisher Scientific, Australia) of an aliquot, indicated the presence of the pathogen. Where ambiguities were observed, the amplicons were then purified using ExoSAP-IT (USB Corporation Cleveland, Ohio, USA) according to the manufacturer’s instructions and sent to the Ramaciotti Centre for Gene Function DNA sequences were determined at the University of NSW using an ABI PRISM 3700 DNA Analyser (Applied Biosystems Inc., Foster City, California, USA). Sequence comparison was made by BLAST analysis with vouchered *Phytophthora* sequences in *Phytophthora*-ID 2.0 (http://phytophthora-id.org/).

### Data analysis

The effectiveness of each hygiene treatment at removing *P. cinnamomi* from boots was assessed using generalised linear mixed effects models (binomial family) with the outcome (1 = positive, 0 = negative) being the response variable. Each boot was regarded as a replicate. The hygiene treatment, including no hygiene (i.e. control), was the fixed effect. Inconsistencies were observed for volunteer gender (classed as male or female) and age (over or under 60), hence each of these factors was included as a random effect in the analysis to account for differences in people’s ability to perform the hygiene treatment and the potential lack of independence in pairs of boots. The 95% profiled confidence interval of each effect was determined and contrasted with the control (no treatment). Other potential random effects (e.g. shoe size, date of each trial or the environmental conditions pertaining on each date) could not be added to the model without producing singular fits. In addition, as most treatment/soil type trials were performed on different dates, the outcomes were highly correlated with date; thus, we make the assumption that the date and the environmental conditions of each trial did not influence the results. To avoid model overfitting, the effect of shoe size on the outcome was assessed separately using a Chi-square test.

The significance of differences in the number of brushes and sprays applied by volunteers within experiments was determined using t tests. The effects of the order of replicates for treatments involving repeated use of fixed brushes in experiments 2 and 3 was assessed in randomisation tests. Positive results were first assigned the replicate number and those numbers were summed to give an observed value. An equal number of positive results were then randomly assigned to replicates 99 times and compared with the observed value. A significant effect of replicate order would be where the observed value was higher than 95 of the randomised values. This is a one-sided test because we hypothesise that the probability of positive results will increase during the experiment. There were too few positive results with the foot bath in experiment 2 to evaluate the effect of replicate order. Analyses were performed in R^47,48^ and graphs were generated with ggplot2 in R^49^.

All analysed data generated during this study are included in this article. All raw datasets used for the analysis are available from the corresponding author on reasonable request.

### Informed consent

Explicit and informed consent from each volunteer involved in the experiments was obtained.

## Results

None of the negative control boots (those tested prior to the experiments) tested positive for *P. cinnamomi*. The effect of shoe size (size 9 or 10) did not significantly affect the likelihood of boots picking up *P. cinnamomi* in inoculated (control) soil (χ^2^ = 0.39, P > 0.05).

### Hygiene metrics and volunteer observations

In the preliminary trial of Equipment 1, where volunteers were asked to brush their boots without removing them, two volunteers were unable to reach the bottom of their boots and therefore could not perform the hygiene task. Most volunteers could not easily check whether the boots were free of soil after brushing.

For equipment 1 (with boots removed), the volunteers spent significantly more time brushing boots with contaminated wet soil (77 ± 3 s) as brushing boots contaminated with dry soil (34 ± 1 s, P < 0.001; Table 1). They also applied significantly more squirts of disinfectant with contaminated wet soil (8.6 ± 0.5 squirts) than with dry soil (6.9 ± 0.2 squirts, P < 0.001), suggesting that they were using it as a drench. The volunteers applied significantly fewer sprays after brushing compared with spraying alone (brushing + spraying = 7.4 sprays ± 0.2, spraying alone = 9.5 sprays ± 0.5; P < 0.001, as determined in a t test) but there was no significant difference in the time taken for brushing when that was or was not followed by spraying (brushing + spraying = 59 ± 3 s, brushing alone = 57 ± 4 s; P > 0.05, as determined in a t test).

**Table 1:** Mean ± SE numbers of sprays, time spent brushing with a handheld brush and number of brushes with a fixed brush by volunteers in experiments involving three types of equipment and four soil/moisture types. Within each treatment for each equipment, the significance of differences in means between loam and sand OR wet and dry soil types was determined by *t* tests; a significant difference is indicated by *** (P < 0.001).

Equipment	Soil and moisture	Sprays (no.)	Handheld brush (s)	Fixed brush (no. of brushes)
1 (Field Hygiene Kit)	Loam (wet and dry)	7.5 ± 0.5	56 ± 3	
	Sand (wet and dry)	7.9 ± 0.2	55 ± 3	
	Wet (loam and sand)	8.6 ± 0.5***	77 ± 3***	
	Dry (loam and sand)	6.9 ± 0.2	34 ± 1	
2 (Fixed Brush and Footbath)	Loam (wet and dry)			19 ± 1
	Sand (wet and dry)			18 ± 1
	Wet (loam and sand)			23 ± 1***
	Dry (loam and sand)			15 ± 1
3 (Commercial Hygiene Station)	Loam (wet and dry)	4.9 ± 0.8		
	Sand (wet and dry)	3.7 ± 0.6		
	Wet (loam and sand)	2.5 ± 0.2		
	Dry (loam and sand)	6.2 ± 0.9***		

For equipment 2, the volunteers rarely checked whether the boots were clean after brushing before either taking them off or stepping in the footbath. Significantly more brushing was applied in wet than in dry soil (23 ± 1 brushes in wet soil and 15 ± 1 in dry soil; P < 0.001).

For equipment 3, there were marked differences in the way the station was used. Some volunteers checked whether boots were clean before stepping off the station, repeating the procedure if they were not, but most did not. The number of sprays resulting in a positive test result for a boot ranged from 1 to 14 (mean = 2.9 ± 0.4). Unlike equipment 1, significantly more sprays were applied to boots with dry soils than boots with wet soils (P < 0.001). This was attributable to a single volunteer who was present for the dry soil tests only; excluding those values, the mean number of sprays in dry soil was 3.5 ± 0.6 and the means were no longer significantly different.

### Effectiveness of hygiene equipment

**Figure 10:**
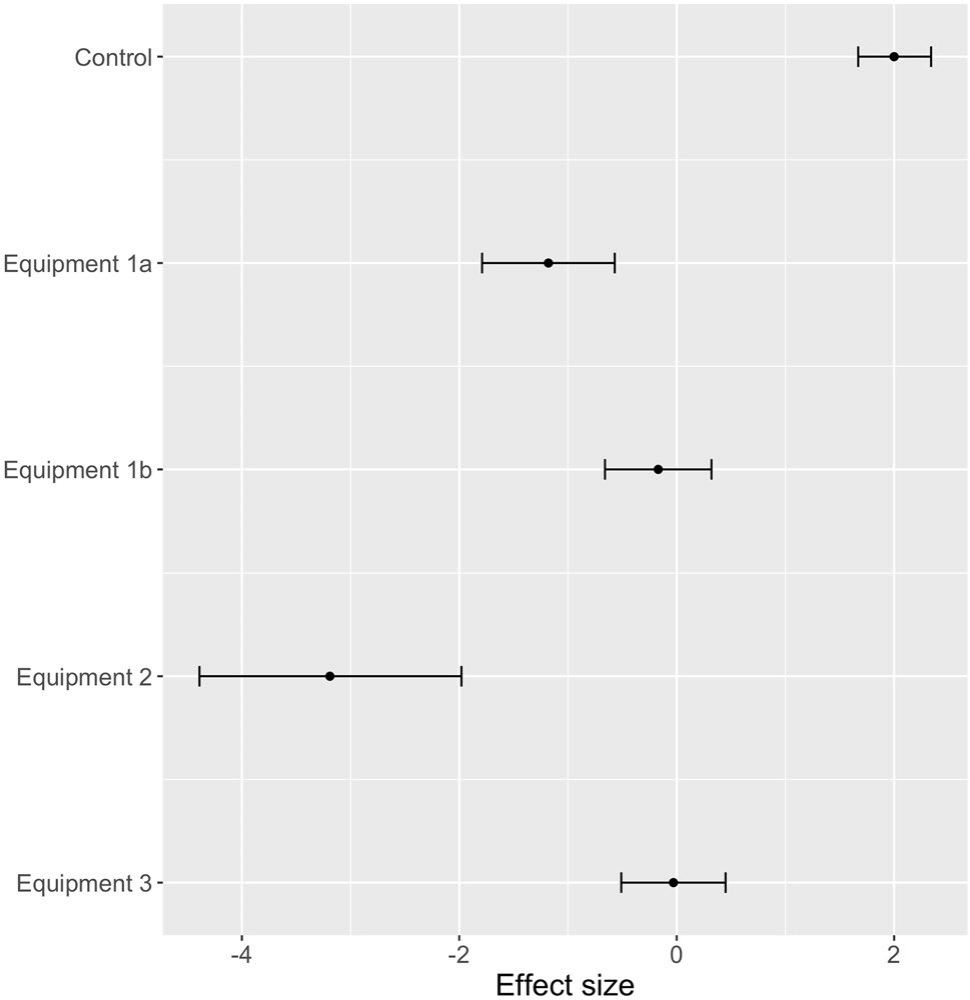
Effect size (estimate and 95% confidence interval of generalised linear mixed models) for the hygiene equipment tested across all soil and moisture types: Control = no hygiene; Equipment 1 = handheld brush + disinfectant (a = 70% methylated spirits, b = 2% BAC); Equipment 2 = Fixed boot brush and footbath with 10% BAC; Equipment 3 = hygiene station with fixed brush and 10% BAC spray; 95% confidence intervals that do not overlap can be considered significantly different. Positive effect values indicated that treated boots retained *P. cinnamomi*.

Comparing the generalised linear mixed effects models (binomial family) for the three pieces of hygiene equipment, the use of hygiene was significantly better than using no hygiene but equipment 2 (a fixed brush followed by a 10% BAC footbath) was the most effective equipment for removing *P. cinnamomi* (Fig. 10). Brushing followed by spraying with 70% methylated spirits (equipment 1a) was significantly better at removing *P. cinnamomi* than equipment 3 (hygiene station with a fixed brush and a 10% BAC spray) but not significantly better than equipment 1b (brushing with a handheld brush followed by spraying with 2% BAC). For equipment 3, the number of sprays of BAC and the area of sole covered by BAC did not apparently influence the outcome: number of sprays (correlation coefficient r = − 0.26, P > 0.05); % of the sole covered by BAC (correlation coefficient r = 0.01, P > 0.05). For equipment 2, the order of brushing boots did not influence the outcome, although the effect was near significant (P = 0.10 as determined in randomisation tests). For experiments involving equipment 3, the order of brushing did not influence the outcome (P = 0.23).

#### Effect of soil type and moisture of hygiene effectiveness

**Figure 11:**
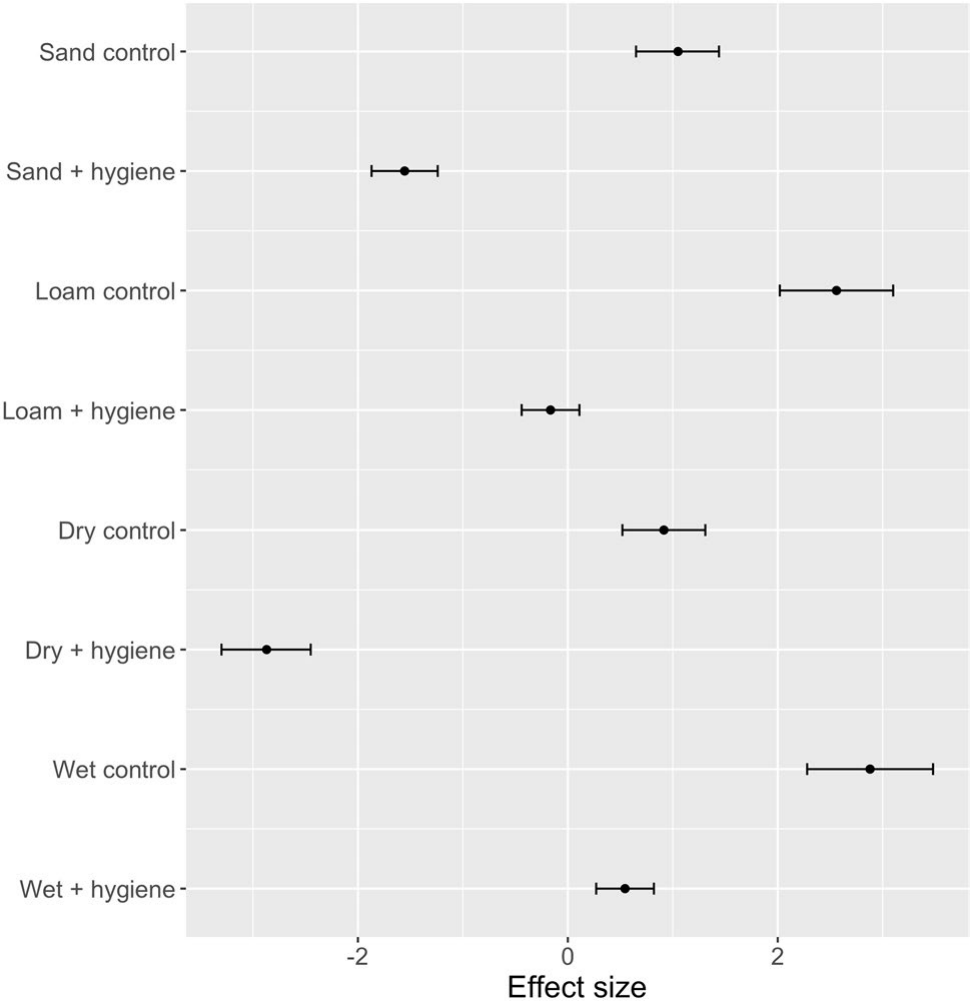
Effect size (estimate and 95% confidence interval of generalised linear mixed models) for soil type (sand or loam) and moisture (wet or dry), with or without hygiene; 95% confidence intervals that do not overlap can be considered significantly different. Positive effect values indicated that treated boots retained *P. cinnamomi*.

The generalised linear mixed models for soil type and moisture with and without hygiene showed that hygiene measures could be effective in either soil and wetness types (i.e. hygiene measures had a significant negative influence on *P. cinnamomi* retention on boots compared with the same soil or moisture type without hygiene) but are likely to be most effective in dry or sandy soil (Fig. 11). Comparing control samples, boots were significantly more likely to pick up *P. cinnamomi* in wet or loam soils than in dry or sandy soils.

### Success rate

**Figure 12:**
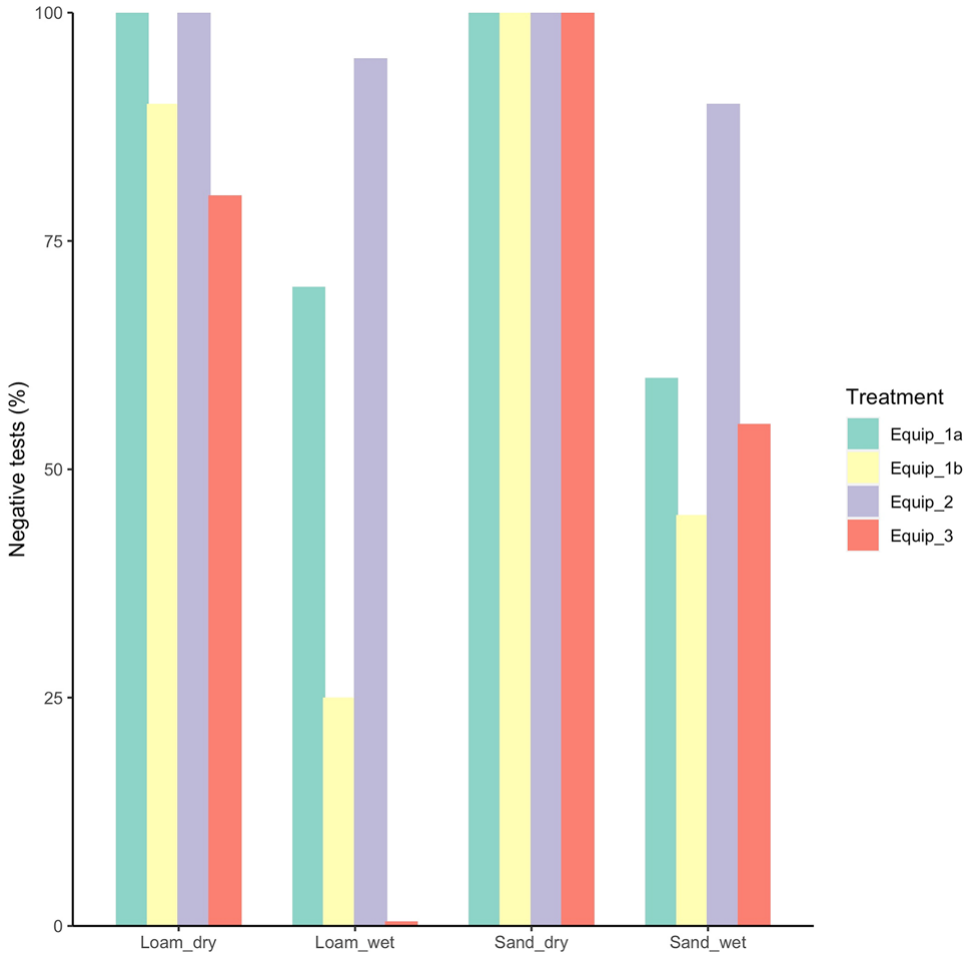
Success rate (% of negative tests, n = 20) of the equipment tested for each of the soil/moisture regimes. Equip_1 = handheld brush + disinfectant (a = 70% methylated spirits, b = 2% BAC); Equip_2 = fixed boot brush and 10% BAC footbath; Equip_3 = hygiene station with fixed boot brush and 10% BAC spray. The zero value was changed to 0.5 to aid with the visual display of data.

The success rate (i.e. the percentage of replicates for which *P. cinnamomi* was not detected on boots after hygiene treatment) was less than 100% in wet soils across all equipment trialled. All equipment was 100% effective in dry sandy soil and equipment 1a and 2 were 100% effective in both dry soil types (Fig. 12). Equipment 3 did not remove *P. cinnamomi* from boots in wet loam soil.

### Effective components of equipment 1

**Figure 13:**
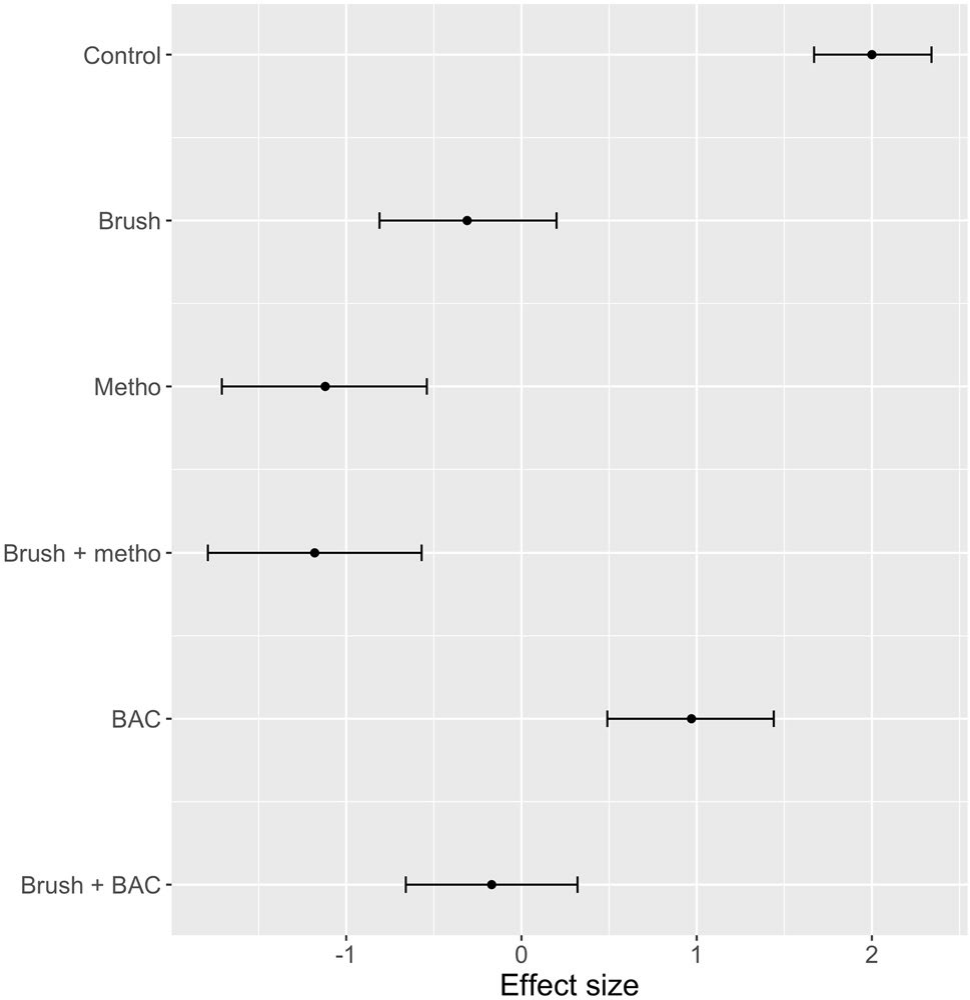
Effect size (estimate and 95% confidence interval of generalised linear mixed models) for components of Equipment 1 across all soil and moisture types: Control = no hygiene; Brush = brushing with a handheld brush; Metho = spraying with 70% methylated spirits; BAC = spraying with 2% BAC; 95% confidence intervals that do not overlap can be considered significantly different. Positive effect values indicated that treated boots retained *P. cinnamomi*.

All components of equipment 1 (a handheld brush, 70% methylated spirits spray, 2% BAC spray) were better at reducing *P. cinnamomi* on boots than doing nothing (Fig. 13). However, brushing or using 70% methylated spirits were the best treatments within equipment 1; doing both was not significantly different from doing either. Spraying with 2% BAC was less effective than brushing and there was no benefit from spraying with 2% BAC after brushing.

### Effective components of equipment 2

**Figure 14:**
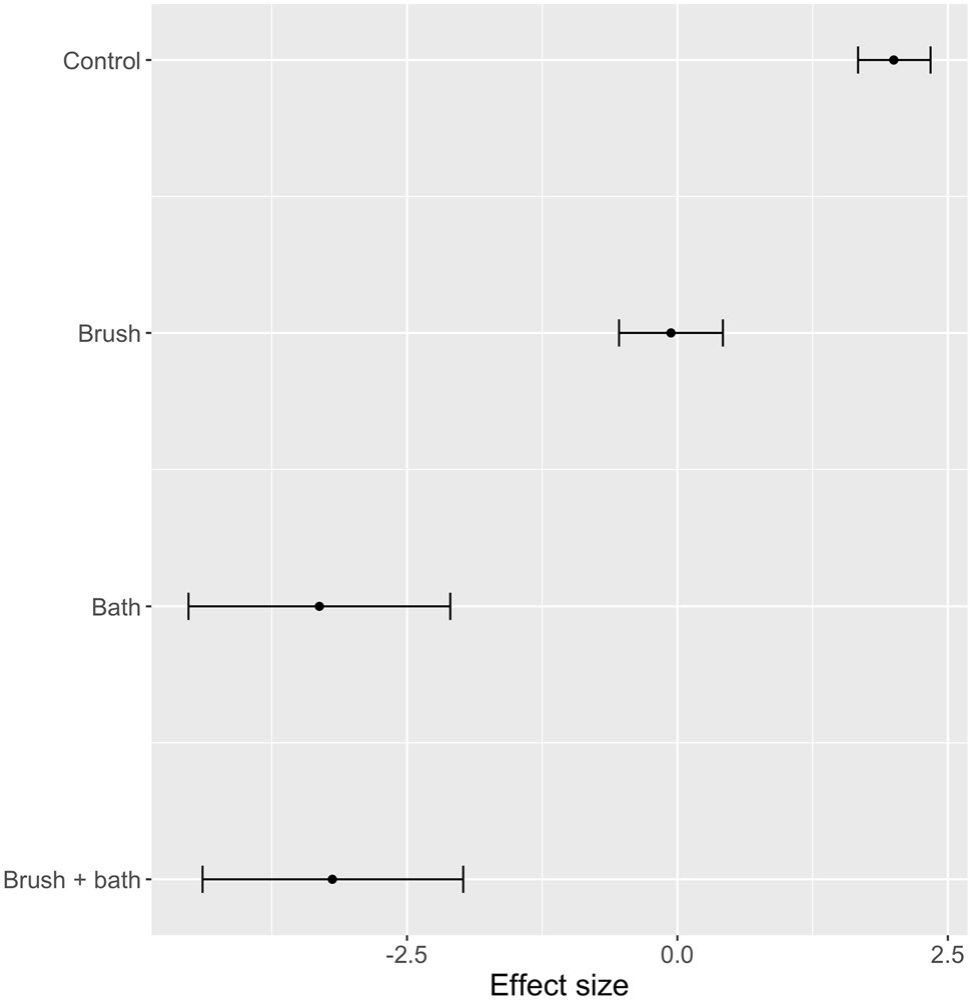
Effect size (estimate and 95% confidence interval of generalised linear mixed models) for components of Equipment 2 across all soil and moisture types: Control = no hygiene; Brush = brushing with a set of fixed boot brushes; Bath = immersing the soles of boots in 10% BAC; 95% confidence intervals that do not overlap can be considered significantly different. Positive effect values indicated that treated boots retained *P. cinnamomi*.

All components of equipment 2 (a fixed boot brush, a 10% BAC footbath or both) were significantly better at removing *P. cinnamomi* from boots than doing nothing (Fig. 14). However, there was no extra benefit from brushing compared with the bath alone.

### Additional experiments

#### BAC concentration

For both concentrations of BAC applied with a spray bottle (5% and 10%), the success rate was 0%, whereas for both concentrations of BAC in a footbath (5% and 10%), the success rate was 100%. These recommended concentrations in the product label were only effective when used in a footbath, rather than as a spray in our experiments, indicating perhaps the importance of cleaning surfaces prior to disinfecting and ensuring sufficient amount and time for the disinfectant to soak into the soil when spraying.

#### Field trial

Of the 20 soil samples collected at potentially infested locations at Nunnock Swamp Nature Reserve, five tested positive for *P. cinnamomi*. The soil residue from three boots and from three of the five locations where *P. cinnamomi* was found to be present from the soil sample tests, tested positive for *P. cinnamomi*.

## Discussion

The hygiene measures we tested and, which are widely used for minimising the risk of *Phytophthora cinnamomi* spread by people in natural vegetation in Australia, were all effective in reducing the number of detections of the pathogen when compared with doing nothing. However, some measures were much less effective under particular soil and moisture conditions. Tjosvold et al*.*^44^ similarly found that *P. ramorum* was picked up by boots in wet conditions but not after walking through dry soil. In our trials, boots that had trodden in wet soils (both sand and loam) were hard to clean—none of the volunteers was able to remove all traces of soil in the time they thought was reasonable for brushing boots; between 14 and 135 s was spent cleaning each boot in contact with wet soil.

A portable hygiene kit, comprising the components of equipment 1 in our trials, is widely recommended as a phytosanitary measure for visitors to natural areas at risk from pathogens, especially where formal hygiene stations are not provided (e.g.^27,50,51^). Based on our experiments, for *P. cinnamomi* the kit need only contain a spray bottle with 70% methylated spirits or a brush; BAC should not be used as a spray at 2% for the purpose of disinfecting boots. Given that the supply of methylated spirits might run out from use or leakage, we recommend both a brush and spray bottle with 70% methylated spirits be carried in a hygiene kit. The brush should also be sprayed with 70% methylated spirits after use.

Hygiene equipment comprising a fixed brush and 10% BAC footbath (equipment 2) will be especially effective at preventing the dispersal of *P. cinnamomi* by hikers. While the brush did not improve the outcome compared with using a footbath alone, it is probably still an important component of the equipment because it reduces the amount of soil that ends up in the bath, and thus the maintenance requirement of the equipment. The efficacy of 5% BAC in addition to the recommended 10% BAC means that managers could use a lower concentration to lower cost and a reduction in concentration over time through dilution from rainwater need not reduce the effectiveness of the apparatus. The near significance of the order of brushing in our experiments suggests that keeping the brushes clean where brushing is not followed by a footbath could be important in reducing the build-up of potentially infested soil. Boots were immersed in the footbath for 30 s in our experiment and we did not test whether shorter times are also effective. Until that is done, signage at hygiene stations with footbaths should indicate that 30 s is the desired time of immersion for each boot.

The hygiene station with a fixed brush and 10% BAC spray (equipment 3) was better than doing nothing but similar in effect to a handheld brush and spraying with a 2% BAC solution from a spray bottle. Places with loamy soil that are relying on equipment such as this for preventing spread of *P. cinnamomi* should consider closing hiking trails in wet conditions. Alternatively, replacing BAC with 70% methylated spirits should be considered if that is practicable and permissible.

The failure of BAC at any concentration as a spray in contrast to its success when used as a bath is curious. On average, volunteers were applying about 10 mL of 2% BAC to cover the entire surface of the boot tread, and in our additional experiment 5% and 10% BAC was applied at a similar volume but with fewer replicates; that is, as a spray alone, none of the concentrations we tested was effective. After either spraying and bathing, the BAC would have remained on the boots for up to five minutes until the soil residue was removed from boots and placed in deionised water for testing, and so the time in contact with BAC is unlikely to be the reason for the contrasting results. The difference may lie in the degree of contact between disinfectant and soil. Although boots were sprayed so that the entire surface was covered, it is possible that the disinfectant did not reach soil deep in the tread. In the bath on the other hand, the disinfectant may have been more likely to saturate the soil in boot treads and kill the pathogen. Whatever the reason, the difference between spray and bath for BAC was profound and should be an important consideration in the use of this disinfectant for hygiene purposes.

Compliance for the success of a hygiene program was not a focus of our study but has been found to be highly variable, from as low as 25–45% of people using supplied hygiene equipment (depending on the method of data capture, Nick Gill, University of Wollongong, pers. comm.) to around 90%^52^. There are many elements that can lead to high compliance levels; e.g. simple but emphatic signage^53^, and continual monitoring of usage and improvement of design^52^. Barriers to cleaning can include perceived difficulties in using equipment and the time required if there are many hikers involved^54^, and lack of awareness about the value in using hygiene measures^26^. For maximal compliance it is also important to devise procedures that are practical for hikers to perform. For instance, a handheld brush in a hygiene kit or fixed to a seat along a hiking trail will be ineffective if hikers cannot easily reach the soles of their boots (as we found with two volunteers) and do not take their boots off; if the signage requests hikers to take their boots off, a seat should be provided for that to happen. In addition, we found that the volunteers rarely checked the soles after brushing when using a fixed brush (and we have observed this in the field). If the primary hygiene measure is brushing, signage should request hikers to check the cleanliness of their boots before proceeding or provide a mirror for the task, as is done with a device in New Zealand^52^.

Bellgard et al*.*^40^ found that a 10% spray with BAC was effective against *P. agathidicida* by killing mycelium and zoospores, in contrast to our findings for *P. cinnamomi*. This suggests that hygiene measures are not necessarily universally effective and may have to be tailored to the pathogen of concern. Our results therefore will be applicable to most situations in Australia, where *P. cinnamomi* is the primary pathogenic threat, but not necessarily to the disease of *Araucaria* spp. in Queensland, possibly attributable to *P. multivora*^55^; further testing is recommended in that case.

Hygiene measures involving brushes and disinfectants in various forms are also utilised other than in native vegetation communities and natural parklands worldwide; these include production systems such as agricultural and forest nurseries, where *P. cinnamomi* (as well as other *Phytophthora* species) is also a pathogen on a wide range of host species. Although our focus is on the protection of native vegetation, the experimental findings and discussion here are highly relevant to a wider audience and scenarios.

Our experiments have shown that some of the hygiene procedures currently recommended for preventing the spread of *P. cinnamomi* and assumed to be effective are ineffective in some circumstances. Equipment 2 (a fixed brush followed by a 10% BAC footbath) may be the best choice for preventing spread to biodiversity assets of great value but, because of its cost, is probably impractical for broadscale use. A portable hygiene kit containing a stiff brush and spray bottle with 70% methylated spirits should be recommended for hikers where management aims to limit the spread of *P. cinnamomi* and other equipment is not provided.

## Supplementary Information

Supplementary Information.

## Data Availability

The raw data for the experiments are available as a Supplementary Data File.
